# Hematologic Outcomes of COVID-19 Patients with and without G6PD Deficiency: A Comparative Study

**DOI:** 10.5339/qmj.2022.54

**Published:** 2022-11-16

**Authors:** Kamran Mushtaq, Ashraf T. Soliman, Abdulqadir J. Nashwan, Fatima Iqbal, Ahmed A. Karawia, Doaa H. Ahmed, Yousef M. Hailan, Mouhammad J. Alawad, Muhammad Abubakar, Mohammadshah I. Gul, Fateen Ata, Mohammed N. Seijari, Mohammed E. Elhadi, Samer J. Kaspo, Mohamed A. Yassin

**Affiliations:** ^1^Department of Gastroenterology and Hepatology Hamad Medical Corporation, Doha Qatar Email & ORCID ID: FAta@hamad.qa & https://orcid.org/0000-0001-7121-8574; ^2^Harvard TH Chan School of Public Health, Boston, USA; ^3^Department of Paediatrics. Hamad Medical Corporation (HMC), Doha, Qatar; ^4^Nursing Department, Hamad Medical Corporation, Doha, Qatar; ^5^Clinical Pharmacy Department, Communicable Diseases Center, Doha, Qatar; ^6^Clinical Pharmacy Department, Hamad Medical Corporation, Doha, Qatar; ^7^Hematology Lab, Hamad Medical Corporation, Doha, Qatar; ^8^Department of Internal Medicine, Hamad Medical Corporation, Doha, Qatar; ^9^Department of Hematology and Oncology National Center for Cancer care and research (NCCCR), Doha, Qatar

**Keywords:** G6PD, COVID-19, SARS-CoV-2, white blood cells, absolute neutrophil count

## Abstract

Introduction: Patients with multiple comorbidities who have coronavirus disease 2019 (COVID-19) have high morbidity and mortality. Glucose-6-phosphate dehydrogenase (G6PD) deficiency has been shown to have an enhanced effect on coronavirus in an earlier study.

Methods: We conducted this comparative observational study to evaluate the effects of COVID-19 disease on G6PD deficiency based on the hematologic parameters, COVID-19-related hospitalizations, and mortality in the state of Qatar between January 2020 and May 2020 at four designated COVID-19 facilities. We identified 41 patients with G6PD deficiency who had documented COVID-19 infection. We compared the results with 241 patients with COVID-19 infection who tested negative for G6PD deficiency**.:** Results: Comparing the COVID-19 positive G6PD deficient with COVID-19 positive G6PD normal activity showed that G6PD normal group had higher white blood cell count (WBC), absolute neutrophil count (ANC), lymphocytes, eosinophils, and monocytes counts versus the G6PD deficient group (*p* < 0.001).

Conclusions: When compared with COVID-19 patients with normal G6PD, patients with COVID-19 infection and G6PD deficiency had lower total WBC, ANC, lymphocyte, monocyte, and eosinophil counts. However, no evidence of increased hemolysis, thrombosis, morbidity, or mortality was observed in COVID-19 patients with G6PD deficiency**.**

## Introduction

Coronavirus disease 2019 (COVID-19) caused by severe acute respiratory syndrome coronavirus 2 (SARS-CoV-2) infection has led a global pandemic with approximately 600 million cases and has already cost nearly 6.4 million lives worldwide since its first description in December 2019 until August 19, 2022.^1,2^ COVID-19 has posed unprecedented challenges to not only healthcare services but to humankind globally. COVID-19 disease causes a respiratory infection, which can result in acute respiratory distress syndrome requiring mechanical ventilation and intensive care management.^3^ High mortality in COVID-19 is associated with several risk factors including age, presence of hypertension, diabetes, coronary artery disease, pulmonary disease, and metabolic syndrome.^4–9^ Most commonly, patients may have respiratory symptoms, which can range from mild to moderate and sometimes progress to severe respiratory distress requiring mechanical ventilation and critical care.^3,10,11^ Understanding COVID-19 disease has an immediate impact globally and has prompted researchers to explore and elucidate the pathophysiology of the disease, as it will allow for better therapeutic targets as well. One hypothesis to explain higher mortality and worse outcomes was possible glucose-6-phosphate dehydrogenase (G6PD) enzyme deficiency.^12,13^

One of the most common enzyme deficiencies in the world is G6PD deficiency, which affectes over 400 million people globally, and it is an X-linked inherited disorder.^14^ G6PD deficiency has a significantly high prevalence in Middle Eastern countries.^15–17^ Either a person can inherit or acquire an enzyme deficiency. Aging and conditions like metabolic syndrome can contribute to acquired G6PD deficiency.^18^ Normal G6PD enzyme is crucial to maintaining redox homeostasis. It has been speculated that viral infection, such as COVID-19 may cause excessive oxidative stress, and patients with G6PD deficiency may not be able to handle the excessive oxidative stress making them more vulnerable to cytopathic effects of the viruses and resulting in worse clinical outcomes, such as hemolytic crisis, hospitalization, and even mortality.^18^

Wu et al. showed that G6PD deficiency can increase the risk of coronavirus infection.^19^ This study has been the basis of the hypothesis that a higher prevalence of disease in these patients might be due to G6PD deficiency. G6PD patients can develop hemolysis after exposure to certain agents, such as drugs.^14^ Several case reports suggest higher mortality and/or hemolytic crisis with COVID-19 infection especially when they had therapies, such as hydroxychloroquine.^20–23^

There are certain repurposed drugs, such as hydroxychloroquine, which are used in SARS-CoV-2 infection and may increase the risk of hemolysis in G6PD deficient patients during COVID-19 management.^24^ Several hypotheses suggest that patients with G6PD may have worse clinical outcomes. It is unclear whether there is any difference in clinical outcomes in patients with COVID-19 with or without G6PD deficiency.

We hypothesized that patients with or without G6PD deficiency might have worse hematologic patterns and clinical outcomes, such as hospitalization and mortality, with COVID-19.

## Methods

We conducted a retrospective analysis of adult patients with symptomatic COVID-19 infection (positive reverse transcription–polymerase chain reaction tests for SARS-CoV-2). We classified disease severity based on the World Health Organization classification.^25^ The patients were admitted to one of the COVID-19 designated facilities of Doha, Qatar, such as the Communicable Disease Center, Hazm Mebaireek General Hospital, Mesaieed General Hospital, and Ras Laffan Hospital. More than 300 patients have been admitted to Qatar's designated COVID-19 healthcare facilities centers between January 2020 and May 2020. On admission, these patients were screened for G6PD deficiency. COVID-19 positive patients were divided into two groups: with and without G6PD deficiency. Additionally, COVID-19 negative patients with or without G6PD deficiency from the community were used as control groups.

Inclusion criteria comprised all COVID-19 positive symptomatic adult patients older than 18 years who required hospitalization. Patients under 18 years old, pregnant women, and those with malignancy were excluded. Clinical presentations, laboratory findings, including inflammatory markers, and radiological findings were recorded. Recovery was defined as the resolution of clinical symptoms assessed by clinicians, such as no fever for more than 3 days, improvement of respiratory symptoms with reduced oxygen requirement, and no further need for hospitalization care. The outcomes included hematologic parameters suggestive of hemolysis and mortality, intensive care unit (ICU) admission, and need for mechanical ventilation. Patients with COVID-19 negative who had complete blood count (CBC) performed at a similar time were included as the control group. G6PD deficient patients following in hematologic clinic that had negative COVID-19 and an available CBC result in the study period were included as a comparison group as well. The study was approved by the Institutional Review Board at Hamad Medical Corporation, Doha, Qatar (MRC-01-20-669).

Statistical analysis was performed using statistical packages SPSS 22.0 (SPSS Inc. Chicago, IL) /Excel statistical Pack software package. The analysis of variance was used to compare variables between COVID-19 positive and negative patients with and without G6PD deficiency when data were normally distributed, and Wilcoxon rank-sum test was performed when the data were not normally distributed. A Linear regression equation was used to find a possible correlation between clinical and laboratory variables. Statistical significance was accepted with a *p* < 0.05.

## Results

There were 41 patients identified with confirmed G6PD deficiency who had developed COVID-19. During the same period, 291 patients with G6PD deficiency without COVID-19 were identified. Comparison between these two groups showed no significant difference in the hematologic parameters, such as hemoglobin (Hb), white cell counts, cell differentials, and bilirubin, suggesting that COVID-19 infection did not precipitate hemolysis in these patients.

Further comparisons were made using two more groups. Two hundred forty-four patients with positive COVID-19 infection with normal G6PD activity and 211 patients with negative COVID-19 infection and normal G6PD activity were used as controls.

Comparing the COVID-19 negative G6PD deficient group with COVID-19 negative G6PD normal activity group showed lower Hb and hematocrit and higher bilirubin levels in the G6PD deficient group (Table 1). Comparing the COVID-19 positive G6PD deficient group with COVID negative G6PD deficient group showed that COVID-19 infection significantly increased the total white blood cell count (WBC), lymphocytes count, and absolute neutrophil count (ANC) but did not increase bilirubin level or decreased Hb level or hematocrit (no hemolysis) (Table 1).

While comparing the COVID-19 positive G6PD deficient with COVID-19 positive G6PD, normal activity showed that G6PD normal group had higher WBC, ANC, lymphocytes, eosinophils, and monocytes counts versus the G6PD deficient group.

None of the 41 patients with COVID-19 infection and G6PD deficiency required blood transfusions, assisted ventilation, or ICU admission. During their stay in the hospital, none of them experienced a thrombotic event or died. Their hospital stay was not different compared with G6PD normal patients.

## Discussion

G6PD is an important enzyme in the pentose phosphate pathway and is responsible for the production of nicotinamide adenine dinucleotide phosphate hydrogen (NADPH), which is used by several types of cells in the body and is vital to maintain the balance of free radical oxygen species.^26^ Mutations in more than 125 different genes have been identified to cause a variable degree of deficiency of the enzyme.^27^ Complete enzyme deficiency is very rare and results in in-utero fetal demise.^28^ The clinical manifestations of G6PD deficiency vary from asymptomatic patients to patients with acute hemolytic anemia, chronic non-spherocytic hemolytic anemia, drug-induced hemolytic anemia, favism, and neonatal jaundice.^29^ Several variants are identified and may have varying degrees of manifestations from mild to a significant hemolytic crisis when exposed to oxidative stress.^29^ The Middle East has a high prevalence of people with significant G6PD deficiency.^30^

Viral infections, such as COVID-19, are speculated to increase oxidative stress through different mechanisms.^31^ SARS-CoV-2 causes an imbalance of the antioxidant (AO) system toward the pro-oxidant system causing increased oxidative stress.^18^ G6PD is essential for an adequate immune response. Both G6PD deficiency and SARS-CoV-2 compromise the AO system through the same pathways rendering G6PDd the Achilles’ heel for COVID-19. In the absence of a functional G6PD enzyme, viral replication becomes easier in several viral infections.^32,33^ Molecular studies have shown that a protein named HSCARG, which is an NADPH sensor and negative regulator of nuclear factor kappa light chain enhancer of activated B cells (NF-κB), gets upregulated in G6PD deficient cells resulting in a decreased NADPH/NADP+ ratio. This causes impairment of NF-κB signaling and enhanced viral replication.^34^ In addition, COVID-19 may enhance the risk of thrombosis and hemolysis in the G6PD deficient patients.^35^

Unfortunately, G6PD markedly decreases with aging, which may lead to an increased morbidity rate in patients with COVID-19 infection.^30^ In addition, it is suggested that metabolic syndrome causes acquired G6PD deficiency and is involved in poor outcomes in COVID-19 patients.^18^ There is high prevalence of venous thromboembolic events in patients with COVID-19, which requires anticoagulation, therefore knowing the hemolytic status of patients is crucial.^36^

In our study, comparing the COVID-19 positive G6PD deficient group with COVID-19 negative G6PD deficient group showed that COVID-19 infection increased significantly the total WBC, lymphocytes count, and ANC but did not increase bilirubin level or decreased Hb level or hematocrit (no hemolysis). However, comparing the COVID-19 positive G6PD deficient with COVID-19 positive G6PD normal activity showed that G6PD normal group had higher WBC, ANC, lymphocytes, eosinophils, and monocytes counts versus the G6PD deficient group. These data suggested a negative effect of G6PD deficiency on the white cell response to COVID-19 infection that may compromise host defense in some patients. Further elucidation in large population-based studies is needed to clarify whether this diminished response may have clinical consequences in any subgroup of the population needs.

None of our 41 patients with G6PD deficiency and COVID-19 infection required assisted ventilation, ICU admission, or blood transfusion. None of them had a thrombotic event or passed away during the hospital stay. Hospital stay was not different compared with patient with normal G6PD. Supporting our findings, another small study that compared 6 patients with G6PD deficiency with 11 patients with normal G6PD reported no major differences except for the lowest PaO2/FiO2 (*p* < 0.05) and lowest Hb levels in the G6PD deficient group.^13^

The strengths of our study include the first relatively large clinical study to investigate the relationship of G6PD deficiency with COVID-19 infection and its impact on relevant clinical outcomes including hemolysis and mortality from a multi-ethnic population. The main limitation of the study is the observational nature of the study and the limitation to hematologic outcomes. In addition, we were unable to identify any high-risk subgroup population due to the small number of COVID-19 infection patients with G6PD deficiency. Further large-scale population-based studies are required to confirm these findings.

## Conclusion

Patients with COVID-19 and G6PD deficiency had lower WBC, ANC, lymphocyte, monocyte, and eosinophil counts compared with COVID-19 patients with normal G6PD. However, no evidence of increased hemolysis, thrombosis, morbidity, or mortality was observed in COVID-19 patients with G6PD deficiency. When clinically possible, drugs known to cause G6PD related issues should not be used until a G6PD diagnostic test has been performed.

## Author Contribution

KM: Methodology, literature review, data collection, analysis, and interpretation, manuscript and writing.

AS, MR, FI, AK, DH, YH, M Alawad, M Abubakar, MG, FA, MS, ME, and SK: Literature review, data collection, and manuscript writing.

MY: Supervision, principal investigator, conceptualization, methodology, critical review, and editing of the manuscript.

All authors: Review and approval of the final manuscript.

## Declarations

### Acknowledgments

None

### Funding

This study did not seek any funding.

### Role of study sponsor or funder

The study sponsor/funder was not involved in the study's design, the collection, analysis, and interpretation of data; writing the report and did not impose any restrictions regarding the report's publication.

### Conflicts of interest/Competing interests

None of the authors have any conflict of interest to disclose.

### Ethics declaration

This work is original, has not been, and is not under consideration for publication in any other Journal. All authors have reviewed and approved the final version of the manuscript. The study was approved by the Medical Research Centre (MRC) Qatar (MRC-01-20-794).

### Consent to participate

Informed consent was not required, as this study was a retrospective data review of medical records.

### Consent for publication

Informed consent was not required, as this study was a retrospective data review of medical records.

### Availability of data and material

Available upon request

### Code availability

Available upon request

## Figures and Tables

**Table 1:** Hematological and bilirubin data of different groups

		Age	WBC	Hb	Hct	MCV	PLT	ANC	Lym^#^	Mon^#^	Eos^#^	Bas^#^	Bili

		year	× 10^9^/L	g/dl	%	fL	× 10^9^/L	× 10^9^/L	× 10^9^/L	× 10^9^/L	× 10^9^/L	× 10^9^/L	μmol^9^/L

COVID positive G6PD deficient N = 41	Mean	46.	6.56	13.37	40.63	87.88	277.12	3.77	2.05	0.56	0.17	0.04	18.19

	SE	1.90	0.47	0.32	0.77	1.26	14.69	0.42	0.11	0.05	0.02	0.00	2.63

COVID negative G6PD deficient N = 291	Mean	51	7.05^&^	13.26	40.97	86.20	263.68	4.15^#&^	2.5	0.64	0.20	0.05	20.95^#&^

	SE	0.65	0.18	0.13	0.46	0.79	8.94	0.16	0.08	0.05	0.02	0.01	1.46

COVID positive G6PD normal N = 244	Mean	50.5	8.7^*^	13.59	40.26	85.67	277.50	5.07^*^	2.20^*^	0.76^*^	0.5^*^	0.05	17.90

	SE	0.83	0.29	0.15	0.43	0.31	6.42	0.28	0.05	0.03	0.03	0.00	1.18

COVID negative G6PD normal N = 211	Mean	48.1	6.72	14.9^#^	43.5^#^	83.61	247.70	3.16	4.0^#^	0.60	0.19	0.14^#^	12.50

	SE	0.86	0.14	0.09	0.33	0.39	4.44	0.14	0.12	0.03	0.01	0.01	0.30

ANOVA (*p* values)		0.01	< 0.001	< 0.001	< 0.001	0.01	0.04	< 0.001	< 0.001	0.03	< 0.001	< 0.001	< 0.001


Abbreviations: WBC: white blood cells, Hb: hemoglobin, Hct: hematocrit, MCV: mean corpuscular volume, PLT: platelets, ANC: absolute neutrophil count, Lymph^**#**^: lymphocyte count. Mon^**#**^: monocyte count, Eos^**#**^: eosinophil, Baso^**#**^: basophil count, G6PD: clucose-6-phosphate dehydrogenase, SE: standard error.

^*^ *p* < 0.05 COVID-19 positive G6PD deficient versus COVID-19 positive G6PD normal activity

^#^ *p* < 0.05 COVID negative G6PD deficient versus COVID-19 negative G6PD normal activity

^&^positive G6PD deficient versus COVID-19 positive G6PD deficient.
